# Growth, morphology, and formation of cinnabarin in *Pycnoporus cinnabarinus* in relation to different irradiation spectra

**DOI:** 10.1007/s43630-023-00493-3

**Published:** 2023-10-28

**Authors:** Christoph W. Schinagl, Bianka Siewert, Fabian Hammerle, Gaja Spes, Ursula Peintner, Michael Schlierenzauer, Pamela Vrabl

**Affiliations:** 1 Department of Microbiology, University of Innsbruck, 6020 Innsbruck, Austria; 2 Department of Pharmacognosy, Center for Molecular Biosciences Innsbruck (CMBI), Institute of Pharmacy, University of Innsbruck, 6020 Innsbruck, Austria; 3 Department of Biotechnology and Food Engineering, MCI-The Entrepreneurial School, 6020 Innsbruck, Austria

**Keywords:** Wood-inhabiting fungi, Natural product, Cinnabarin, Light-induced pigmentation, Standardized irradiation conditions, LIGHT BOX, *Arthroconidia*

## Abstract

**Background:**

The demand for natural pigments in general, and for fungi-derived pigments in particular, is constantly rising. Wood-decomposing fungi represent a promising source for natural pigments and they are usually easy to cultivate in pure culture. One of them, i.e., *Pycnoporus* *cinnabarinus*, offers a highly interesting spectrum of bioactivity, partly due to the formation of the orange–red pigment cinnabarin. However, apart from a few studies addressing its diverse potential biotechnological applications, there is still a large gap of knowledge concerning the influence of light on the formation of cinnabarin. The aim of this work was to investigate the effect of different irradiations on the cinnabarin content, the growth, and the morphology of three different *P. cinnabarinus* strains. We used highly standardized irradiation conditions and cultivation techniques in combination with newly developed methods for the extraction and direct quantification of cinnabarin.

**Results:**

Red, green, blue, and UV-A irradiation (mean irradiance *E*_e_ = 1.5 ± 0.18 W m^−2^) had considerable effects on the growth and colony appearance of all three *P. cinnabarinus* strains tested. The cinnabarin content determined was, thus, dependent on the irradiation wavelength applied, allowing strain-specific thresholds to be defined. Irradiation with wavelengths below this strain-specific threshold corresponded to a lower cinnabarin content, at least at the intensity applied. The orange–red pigment appeared by light microscopy as incrusted extracellular plaques present on the hyphal walls. Highly efficient vegetative propagation occurred by arthroconidia, and we observed the tendency that this asexual reproduction was (i) most frequent in the dark but (ii) never occurred under UV-A exposure.

**Conclusion:**

This study highlights a differential photo-dependence of growth, morphology, and cinnabarin formation in *P. cinnabarinus*. This confirms that it is advisable to consider the wavelength of the light used in future biotechnological productions of natural pigments.

**Graphical abstract:**

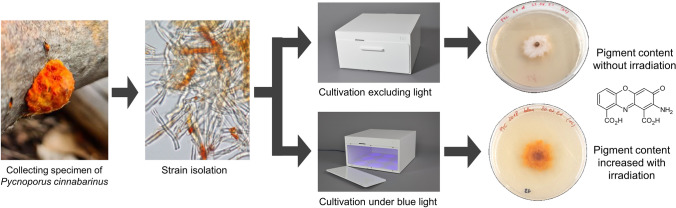

**Supplementary Information:**

The online version contains supplementary material available at 10.1007/s43630-023-00493-3.

## Introduction

Biologically active natural products have been valuable for humankind since ancient times due to their enormous chemical diversity. Since the twentieth century, efforts to study terrestrial organisms other than plants for bioactive natural products have been steadily increasing. In the course of this, more and more attention has been drawn to the kingdom *Fungi*, including fungal endophytes [1] and fungi from marine ecosystems [2]. Although fungal metabolites were traditionally used for manufacturing, preservation or dyeing [3, 4], the modern pharmacotherapeutic use of fungal metabolites, which became possible in the first place through industrial biotechnological production, only began in the middle of the last century. Since then, many antibacterial and antifungal agents derived from fungi have significantly improved modern medicine. In recent decades, the pharmaceutical exploration of light-activated natural products from fungi, i.e., fungal pigments as photosensitizers [5], has become a focus of research interest.

Light is an important factor influencing fungal physiology (e.g., [6, 7]). The perception of light is a widespread ability of fungal organisms, which could be considered as a photo-sensory part of an early warning system for light-induced stress situations [6–9]. Interestingly, the formation of pigments seems to be a light response of fungi and therefore, from a biotechnological point of view, the formation of fungal pigments or secondary metabolites in general is often promoted by the absence [10] or presence of light [11] and may also depend on the wavelength [12]. For the *Monascus* strain M9, it is for example known that continuous blue light irradiation reduced the pigment production, while varying blue light levels caused different changes in the production level of the red, yellow, and orange pigments [13]. In addition, factors such as duration and intensity of illumination and even the direction of the light source may play a role, as shown by the example of the positive phototropism of the sporangiophores of *Phycomyces* *blakesleeanus* [14].

*Pycnoporus* (Basidiomycota, *Polyporaceae*) is a genus of bracket fungi forming bright vermilion, console-shaped annual fruiting bodies on dead wood. The main pigment of these conspicuously colored fruiting bodies is cinnabarin, an amino substituted phenoxazine-3-one derivative [15]. Cinnabarin is known for its bactericidal or bacteriostatic [16, 17], antiviral [18], and antitumor effects [19]. The main environmental factors influencing cinnabarin production are, however, still poorly understood.

Results from studies based on submerged cultures of the tropical Cinnabar bracket fungus *Pycnoporus sanguineus* have already indicated that light may play a key role in pigment formation. Exposure to light increases fungal growth and pigment content, including cinnabarin concentration, compared to darkness [20].

In this study, we were interested in the common Northern Cinnabar bracket fungus *P.* *cinnabarinus*. We were especially interested in fungal strain-dependent light response: is light inducing changes in fungal growth and morphology? Can cinnabarin content be manipulated by different wavelengths of light? This is of particular interest as our previous studies already indicated that *P. cinnabarinus* extracts have promising photochemical activity [21]. Thus, we wanted to understand the irradiation requirements for the formation of photochemical compounds in pure cultures of this bracket fungus.

## Materials and methods

### Chemicals

If not stated otherwise, all chemicals were at least of analytical grade and purchased from Roth (Karlsruhe, Germany). The solvents for the isolation and extraction processes were obtained from VWR International (Vienna, Austria). Distillation of acetone occurred prior to its use. Solvents used in HPLC experiments had at least *pro analysis* (*p.a.*) quality and were purchased from Merck (Merck KGaA, Darmstadt, Germany). Ultrapure water was obtained via the Sartorius arium^®^ 611 UV purification system (Sartorius AG, Göttingen, Germany).

### Instruments

The fungal agar colonies were ground with the mill Retsch^®^ SM 2000 (RETSCH GmbH, Haan, Germany), which was equipped with a 4.0 mm mesh. The weighing instruments KERN ALS 220-4 (KERN & SOHN GmbH, Balingen-Frommern, Germany) and Sartorius Cubis^®^-series (Sartorius AG, Göttingen, Germany) were utilized for weighing in of samples. The rotary evaporator Heidolph LABOROTA 4000-efficient (Heidolph Instruments GmbH & CO. KG, Schwabach, Germany) was used together with a vacuum pump and a vacuum controller for the evaporation of solvents under reduced pressure. Moreover, the ultrasonic baths Sonorex RK 106, Sonorex RK 52, and Sonorex TK 52 (BANDELIN electronic GmbH & Co. KG, Berlin, Germany) were used. The samples were mixed with the vortex mixer Vortex-Genie 2 (Scientific Industries, Inc., Bohemia, New York). Volumes were transferred with pipettes and tips from Eppendorf AG (Hamburg, Germany) and STARLAB International GmbH (Hamburg, Germany). Any other specific instruments are mentioned and described in the respective chapters.

### Strains

*Pycnoporus cinnabarinus* (Jacq.) P. Karst fruiting bodies were collected in the Austrian central alpine area on deciduous trees at altitudes between 650 and 1100 m above sea level (IBF20160231 at c. 650 m, IBF20170021 at c. 1100 m, and IBF20180012 at c. 800 m). Voucher specimens were deposited in the Tiroler Landesmuseum IBF (Index Herbariorum ID 126158, Hall in Tyrol, Tyrol, Austria). Pure culture isolates were obtained for all strains and preserved in the culture collection of the Department of Microbiology, University Innsbruck under the same voucher numbers. The identity was confirmed by morphological hallmarks and barcode sequencing as described previously [22]. The ITS sequences are deposited at the GenBank database (https://www.ncbi.nlm.nih.gov/genbank/, accessed 2023/09/23) under the following GenBank accession numbers: IBF20160321 (OR584232), IBF20170021 (OR584233), and IBF20180012 (OR584234). Cultures were preserved in 10% skim milk and in 40% glycerol at *T* = –80 °C at the Department of Microbiology in Innsbruck (Tyrol, Austria).

### Media and inocula

Preliminary experiments with malt extract agar (MEA), potato dextrose agar (PDA), and Sabouraud dextrose agar (SDA) supplemented with 2% dextrose revealed MEA as best medium for both growth and pigment production. For the preparation of MEA, malt extract (*c* = 12 g L^−1^, bacteriological, Roth, Germany) and agar (*c* = 19.2 g L^−1^, Kobe I, Roth, Germany) were autoclaved together at *T* = 115 °C for *t* = 15 min. The medium volume (*V* = 20 mL) per Petri dish was applied with a dispenser. Preparation of the inocula was done by withdrawing mycelial discs from just behind the growing margin of the cultures with a sterile, sharpened corkscrew (inner diameter 6.7 mm) to facilitate a more constant starting point for the lag phase compared to the application of spore suspension as inoculum.

### Cultivation

#### Temperature effect on fungal growth: estimation in terms of radial growth

All isolates were incubated for 7 days at *T* = 25 °C and a relative humidity of 60% prior to the experiments. For the determination of cardinal temperatures, the three *P. cinnabarinus* strains were incubated in triplicates under dark conditions (to exclude the factor of light) and at the following ten temperatures: *T* = 10, 20, 25, 30, 32, 35, 37, 40, 42, and 45 °C. After 5 days of incubation, the cultures were photo-documented (supplementary information, Figure S12–15) and the radial growth, i.e., the culture diameter (cm) was measured.

#### Irradiation effect on fungal growth: estimation in terms of dry weight

For the irradiation experiments, cultures were grown at *T* = 25 °C and a relative humidity of 60% during 7 days prior to the experiments. Fungal inocula were placed on a commercial cellophane membrane that had been cut into discs. The diameter of these discs was 4 mm less than the inner diameter of the Petri dishes (i.e., 82 mm), thus enabling a more convenient applicability under the sterile airstream. Estimation of dry weight: see workflow in Section “Sample preparation and extraction”.

### Irradiation

Irradiation was performed with an irradiation device (LIGHT BOX) and its established handling as described previously [23, 24]. In brief, to achieve highly standardized growth and illumination conditions (i.e., homogeneous illumination at defined wavelengths), this ventilated instrument was used within a climatic chamber. Inoculated Petri dishes were immediately incubated in a LIGHT BOX and kept for 7 days at *T* = 25 °C and at 60% relative humidity. The cultures were either kept constantly illuminated or in complete darkness (supplementary information, Figure S15).

### Microscopic evaluation and documentation

All pure culture isolates were examined with standard microscopic techniques using water and cotton blue. Microscopic documentation and measurements were made with a Nikon DS Fi1 camera and the corresponding software NIS Elements 4.13.04 (Nikon Europe, Amsterdam, Netherlands). All measurements were made at 1000-fold magnification with a Nikon Plan Fluor 100X/0.5-1.3 oil immersion objective.

### Sample preparation and extraction

The cultures were processed as follows: first, the cellulose hydrate membrane was carefully peeled off from the solidified medium with tweezers. The mycelium was scraped off the membrane, transferred into a glass vial, and stored in a deep freezer (*T* = –80 °C). After a storage time of approx. 24 h, the frozen sample was subjected to freeze-drying (Benchtop Pro, SP Scientific, Warminster, USA). After the determination of the dry weight, the individual samples were finely ground with mortar and pestle. An aliquot (*m* = 2–8 mg) of the ground sample was extracted with dimethyl sulfoxide (DMSO, *V* = 500 µL) by ultra-sonication (*t* = 5 min), followed by centrifugation (approx. 16 000 rcf, *t* = 10 min). The supernatant was filtered through cotton wool into a 2 mL volumetric flask. This extraction procedure was repeated twice and all supernatants were collected in the volumetric flask. In a last step, the targeted volume (*V* = 2 mL) was complemented with DMSO. The obtained solution was directly subjected to HPLC–DAD analysis.

### High-performance liquid chromatography (HPLC)

The HPLC–DAD experiments were conducted using the modular system Shimadzu LC-20AD XR (Shimadzu Europa GmbH, Duisburg, Germany) equipped with a solvent degasser (DGU-20A3), a pump (LC-20AD XR), an auto-sampler (SIL-20AC XR), a column thermostat (CTO-20AC), and a diode-array detector (SPD-M20A). A Synergi Hydro-RP 80 Å column (150 × 4.60 mm, 4 µm) from Phenomenex (Aschaffenburg, Germany) was used as stationary phase. The mobile phase comprised water with 0.1% v/v formic acid (A) and acetonitrile (B). Elution was performed in gradient mode (0 min: 25% B, 11 min: 53.4% B, 12 min: 95% B, 14 min: 95% B, 15 min: 25% B, 17 min: 25% B, followed by 8 min of re-equilibration with 25% B). Flow rate, sample volume, and column temperature were adjusted to *Q* = 1.2 mL/min, *V* = 10 µL, and *T* = 40 °C, respectively. The DAD was set to *λ*_det_ = 450 nm. The area of the target peak (*t*_r_, cinnabarin = 5.21 min) was integrated with Origin 2020 (OriginLab Corporation, North Hampton, United States), see supplementary information, Figure S1 for a representative run. For further data processing, MS Excel 365 (Microsoft Cooperation, Redmond, USA) and R Studio (2020 1.3.1093, R version 4.0.2 (2020-06-22)) were used.

### Annotation process

UHPLC-HRMS/MS: The UHPLC analysis of a *Pycnoporus* *cinnabarinus* acetone extract (*c* = 2.5 mg/mL, solvent for dissolution = DMSO) was performed on a Vanquish system (Thermo Scientific, Waltham, MA, USA) consisting of quaternary pump, auto-sampler, column oven, and variable wavelength detector connected to a Thermo Scientific Exploris 120 Orbitrap HRMS unit. Separation was carried out on a Waters Acquity BEH C18 column (100 mm × 2.1 mm; particle size 1.7 µm) protected by a SecurityGuard ULTRA guard C18 pre-column. The mobile phase comprised water with 0.1% formic acid (A) and acetonitrile with 0.1% formic acid (B). The applied gradient was as follows: from 1 to 99% B in 9 min, followed by an isocratic step at 99% B for 1 min. Finally, the column was re-equilibrated with the initial solvent composition (i.e., 0% B) for 6 min. The flow rate, column temperature, auto-sampler temperature, and injection volume were adjusted to 0.5 mL/min, 40 °C, 20 °C, and 1 µL, respectively. The detection wavelengths were set to 468 and 519 nm.

The instrument was controlled by Thermo Scientific Xcalibur 4.4 software. Calibration of the mass analyzer was done via the Thermo Scientific proprietary calibration mix and the respective automatic calibration function. The mass spectrometric parameters were as follows: heated-ESI ionization source, positive polarity with static spray voltage (3000 V), sheath gas (N_2_): 37 arbitrary units, auxiliary gas (N_2_): 10 arbitrary units, sweep gas (N_2_): 0 arbitrary units. Temperature of the ion transfer tube and vaporizer was adjusted to 370 and 420 °C, respectively. MS data (range 100–1000 *m*/*z*) were recorded from 0 to 16 min with a resolution of 60 000 FWHM for MS1. Data-dependent experiments were conducted with stepped collision energies (15, 30, and 45 eV) at a resolution of 15 000 FWHM. The number of dependent scans was set to 3. The following selection of filters was employed: intensity threshold filter (5.0E5), dynamic exclusion (parent ions were placed in the exclusion list for 2 s after detection), isotope exclusion, charge state, and apex filter. In addition, a specific exclusion list was created for the measurement using DMSO as a background extract with an IODA Mass Spec notebook [25].

Data processing with MZmine3: The data obtained in positive mode (.raw format, Thermo Scientific) were imported into MZmine3 software [26]. For mass detection at the MS1 level, the noise level was set to 1.0E6. For MS2 detection, the noise level was set to 0.00. The ADAP chromatogram builder parameters were set as follows: minimum group size in number of scans, 4; group intensity threshold, 1.0E6; minimum highest intensity, 1.0E6 and scan to scan accuracy (*m*/*z*) of 0.0030 or 10.0 ppm. The ADAP feature resolver algorithm was used for chromatogram deconvolution with the following parameters: S/N threshold, 30; minimum feature height, 1.0E6; coefficient/area threshold, 110; peak duration range, 0.01–1.0 min; retention time (RT) wavelet range, 0.01–0.10 min. Isotopes were detected using the 13C isotope filter (formerly: isotope grouper) with a *m*/*z* tolerance of 0.0030 or 10.0 ppm, a retention time tolerance of 0.05 min (absolute), the maximum charge set to 1, and the representative isotope used was the most intense. Alignment was done with the Join aligner (*m*/*z* tolerance, 0.0030 or 10.0 ppm; RT tolerance, 0.05 min; Weight for RT, 70) and the aligned list was filtered using the Duplicate peak filter (*m*/*z* tolerance, 0.0030.0 or 10.0 ppm; RT tolerance, 0.10 min). The files were filtered with the Feature list rows filter (RT, 0.00–10.00 min) and only the ions with an associated MS2 spectrum were kept.

SIRIUS: After data processing, the feature corresponding to cinnabarin (RT 3.26 min—UHPLC analysis) was exported to SIRIUS (https://bio.informatik.uni-jena.de/software/sirius/ [27]) using the dedicated function in Mzmine3 with the Merge MS/MS option activated and the *m*/*z* tolerance set to 0.0030 *m*/*z* or 10.0 ppm. Following parameters were used for the SIRIUS molecular formula calculation: possible ionizations: [M + H]^+^, [M + K]^+^, [M + Na]^+^; instrument: Orbitrap, MS2 mass accuracy (ppm): 5 ppm, candidates stored: 10; min candidates per ion stored: 1; use DB formulas only: all. ZODIAC was used with default settings. The CSI:FingerID fingerprint prediction [28] was carried out with subsequent parameters: fallback adducts: [M + H]^+^, [M]^+^, [M + K]^+^, [M + Na]^+^; general: score threshold activated, search DBs: all. Lastly, the CANOPUS compound class prediction was performed [29–31].

### Biomaterial and extraction

Large quantities of *P.* *cinnabarinus* fruiting bodies were collected in Innsbruck (Hungerburg/Hoch-Innsbruck: 47.29° N, 11.40° E, Tyrol (Austria)) in August 2019. The freshly harvested fruiting bodies were thoroughly cleaned with a brush, frozen over night at *T* = − 80 °C, and freeze-dried afterward. The dried fruiting bodies were stored in paper bags at room temperature in the dark until further use.

The dried biomaterial was ground using a laboratory mill (mesh size = 4.0 mm). The highly voluminous powder (*m* = 47.53 g) was successively extracted with petroleum ether (*V* = 1.4 L, *n* = 3), dichloromethane (*V* = 1.3 L, *n* = 3), and acetone (*V* = 1.5 L, *n* = 3) by ultra-sonication (*t* = 10 min each). Extracts were vacuum filtrated (filter: MN615, retention capacity 4–12 µm, Macherey-Nagel GmbH & Co. KG, Düren, Germany) and solvents were removed by rotary vacuum evaporation at *T* = 40 °C. Extract yields were for petroleum ether *η* = 323.4 mg (0.68% DW), for dichloromethane *η* = 444.4 mg (0.93% DW), and for acetone *η* = 355.9 mg (0.75% DW).

### Isolation and characterization of cinnabarin

Based on a previous protocol [32], an optimized isolation process was developed: an aliquot (*m* $\sim$ 30–40 mg) of the acetone extract was covered with 10 mL of acetone. After the sample was partially dissolved using an ultrasonic bath (*t* = 10 min), it was centrifuged (2000 rcf, *t* = 5 min). The supernatant was recovered and transferred into a round-bottom flask. These steps were repeated until the extract was completely dissolved. The solution in the round-bottom flask was diluted with ultrapure water (*q.s.*, approx. *V* = 100 mL) and acetone was evaporated under reduced pressure. As the acetone evaporated, an orange–red precipitate was formed. The acetone-free aqueous solution was vacuum filtered (used filter paper: retention capacity 4–12 µm) and the filter cake washed with hot ethanol (*n* = 4, *V* = 10 mL, *T* ~ 60 °C) and hot dioxane (*n* = 4, *V* = 5 mL, *T* ~ 60 °C). Finally, the precipitate was dried by lyophilization (*η* ~ 2–3 mg (5–10%)).

Cinnabarin (CAS 146-90-70) was obtained as a red solid from the acetone extract of *P. cinnabarinus*. Experimental data were in line with [19]. Furthermore, UHPLC high-resolution MS/MS-based analyses were performed as additional annotation tool confirming the chemical formula C_14_H_10_N_2_O_5_ via i.a. the molecular ion peak [M + H]^+^ at *m*/*z* = 287.0649. All relevant spectra for the classic chemical characterization (Fig S1–S6) and the annotation workflow are displayed in the SI part (Fig S7–11 and Table S1). ^1^H-NMR (400 MHz, DMSO-d_6_, 25 °C): *δ* = 9.60 (brs, 1H, N*H*_α_), 8.74 (brs, 1H, N*H*_β_), 7.55 (*dd*, 1H, *J* = 7.6, 7.5 Hz, C_ar_*H*-7), 7.53 (*dd*, 1H, *J* = 7.7, 2.1 Hz, C_ar_*H*-8), 7.49 (*dd*, 1H, *J* = 7.6, 2.0 Hz, C_ar_*H*-6), 6.63 (s, 1H, C_ar_*H*-4), 5.50 (*t*, 1H, *J* = 5.0 Hz, O*H*), 4.89 (*d*, 2H, *J* = 4.6 Hz, C*H*-12). UV/Vis (MeOH): *λ*_max_ = 202, 233, 430, and 446 nm. IR (cm^−1^) = 3267*w*, 2916*s*, 2849*s*, 1672*w*, 1655*m*, 1591*s*, 1580*s*, 1466*m*, 1302*w*, 1187*w,* 1081*m*, 974*m*, 912*w*, 857*w*, 787*m*, 689*m*, 608*w*, 562*w*, 506*w*, 450*w,*434*w.*

### Calibration curve

Two separately weighed stock solutions in DMSO (*c* = 100 µg/mL) were used to prepare 14 calibration levels (i.e., 7 per stock solution) by dilution in DMSO. The resulting solutions were immediately analyzed by HPLC–DAD as described above. Integration of the target peak was done with Origin 2020 and the calibration curve was calculated using a linear regression in MS Excel 365 (Microsoft Cooperation, Redmond, USA).

The following calibration curve parameters were determined for cinnabarin based on the ICH guideline [33]: (i) regression equation *y* = 391,079.03 *x* – 32.30, (ii) *R*^2^ = 0.9997, (iii) linear range = 2.28 E−4 - 0.11 mg mL^−1^, (iv) LOD = 4.96 E−4 mg mL^−1^, and (v) LOQ = 1.50 E−3 mg mL^−1^.

## Results

### Determining and standardizing the conditions for the irradiation experiments—media and cultivation temperature

**Fig. 1:**
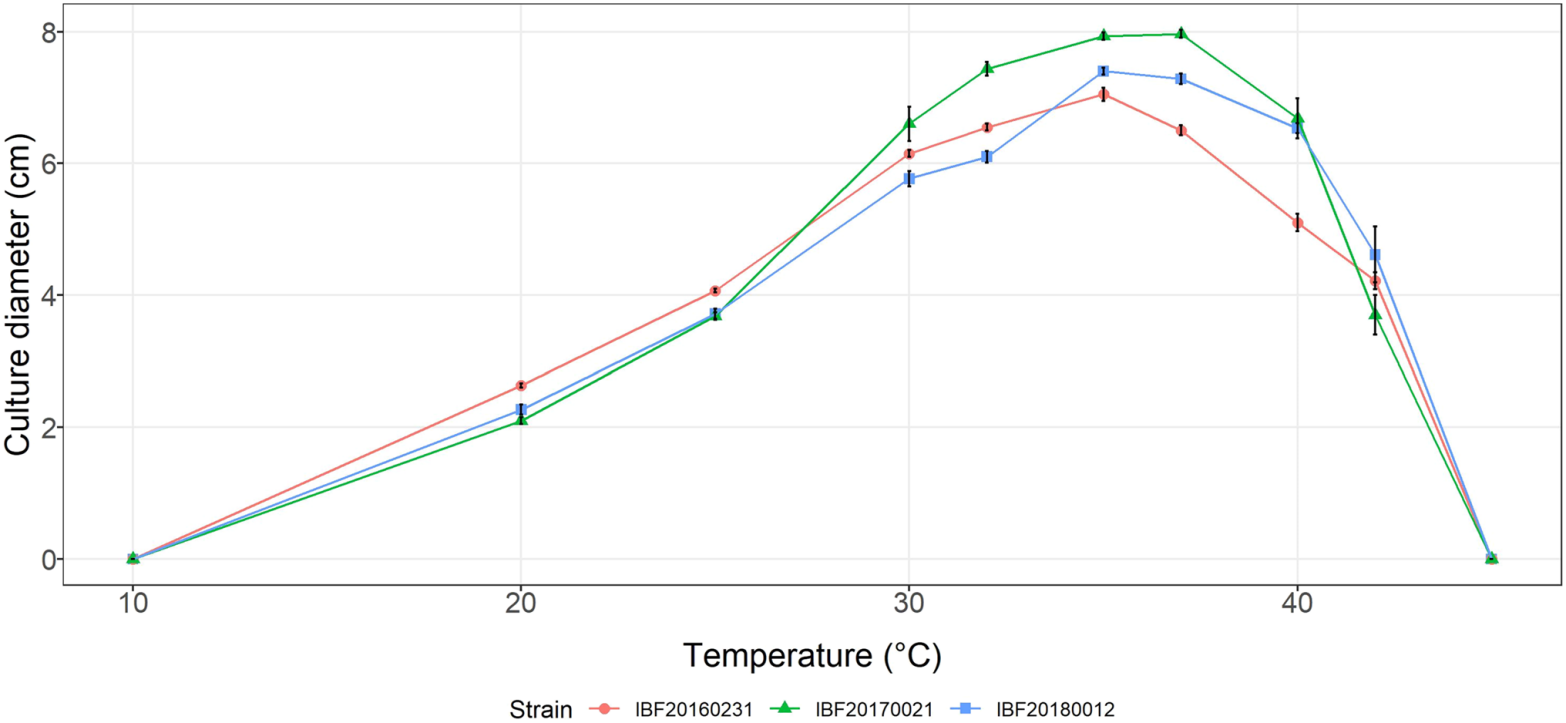
Radial growth (5 days in darkness on MEA) of three *P. cinnabarinus* strains in dependence of the temperature. Means and standard deviations (*n* = 3) of the culture diameters of each strain are shown

Preliminary testing of growth on different solidified media (MEA, PDA, and SDA 2%) revealed MEA as the best match for the growth of *P.* *cinnabarinus* and its red–orange pigmentation. To determine the optimal temperature for growth in the subsequent irradiation experiments, triplicates were cultivated at ten different temperatures and kept in darkness to exclude the factor light (Fig. 1).

All *Pycnoporus cinnabarinus* strains were highly temperature tolerant up to *T* = 42 °C (Fig. 1). The largest colony diameters were observed in a temperature range between *T* = 35–37 °C.

For the subsequent irradiation experiments, a cultivation temperature of 25 °C was chosen (see Fig. 1) based on three rationales. (i) The chosen temperature offered the possibility to detect enhancing effects of irradiation on fungal growth, as the maximum specific growth rate (*µ*_max_) was not reached at T = 25 °C (Fig. 1). (ii) The possibility to detect irradiation-related inhibiting effects on growth in terms of both, culture diameter and biomass, was given, as sufficient biomass was produced. (iii) An even starting point was provided for the estimation of growth under the different irradiation conditions, as the culture diameters between the strains differed only slightly at this temperature.

### Effects of continuous irradiation on biomass and cinnabarin content of different P. cinnabarinus strains

**Fig. 2:**
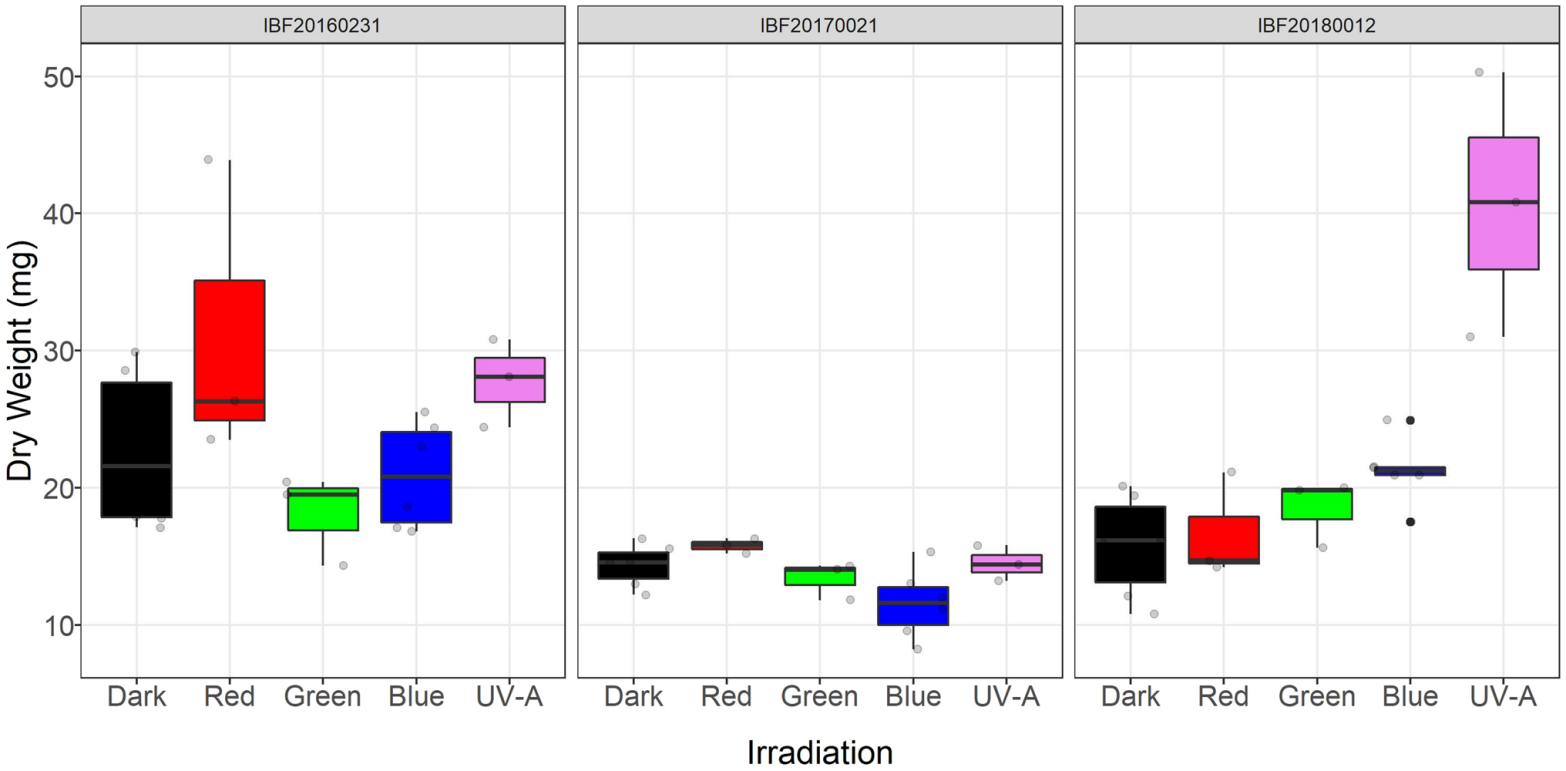
Formation of biomass (mg DW per Petri dish) in cultures of three strains of *P. cinnabarinus* (IBF20160231, IBF20170021, and IBF20180012) grown on MEA (7 days at 25 °C) in dependence of the following continuous irradiation conditions. Wavelength of peak emission, *λ*_peak_, and full width half maximum (FWHM) are indicated in brackets: red (635 ± 18 nm), green (519 ± 38 nm), blue (452 ± 19 nm), and UV-A (369 ± 13 nm). Irradiance for light spectra in the center of the Petri dishes was *E*_e_ = 1.5 ± 0.18 W m^−2^. Dark conditions served as control. *Median* and standard deviation are depicted; the light gray circles represent individual measurements of cultures

Irradiation with different wavelengths showed no consistent effect on the formation of fungal biomass for the three strains (Fig. 2). After 7 days, the biomass yield in terms of dry weight, ranged from *m* = 12 to 55 mg per Petri dish.

**Fig. 3:**
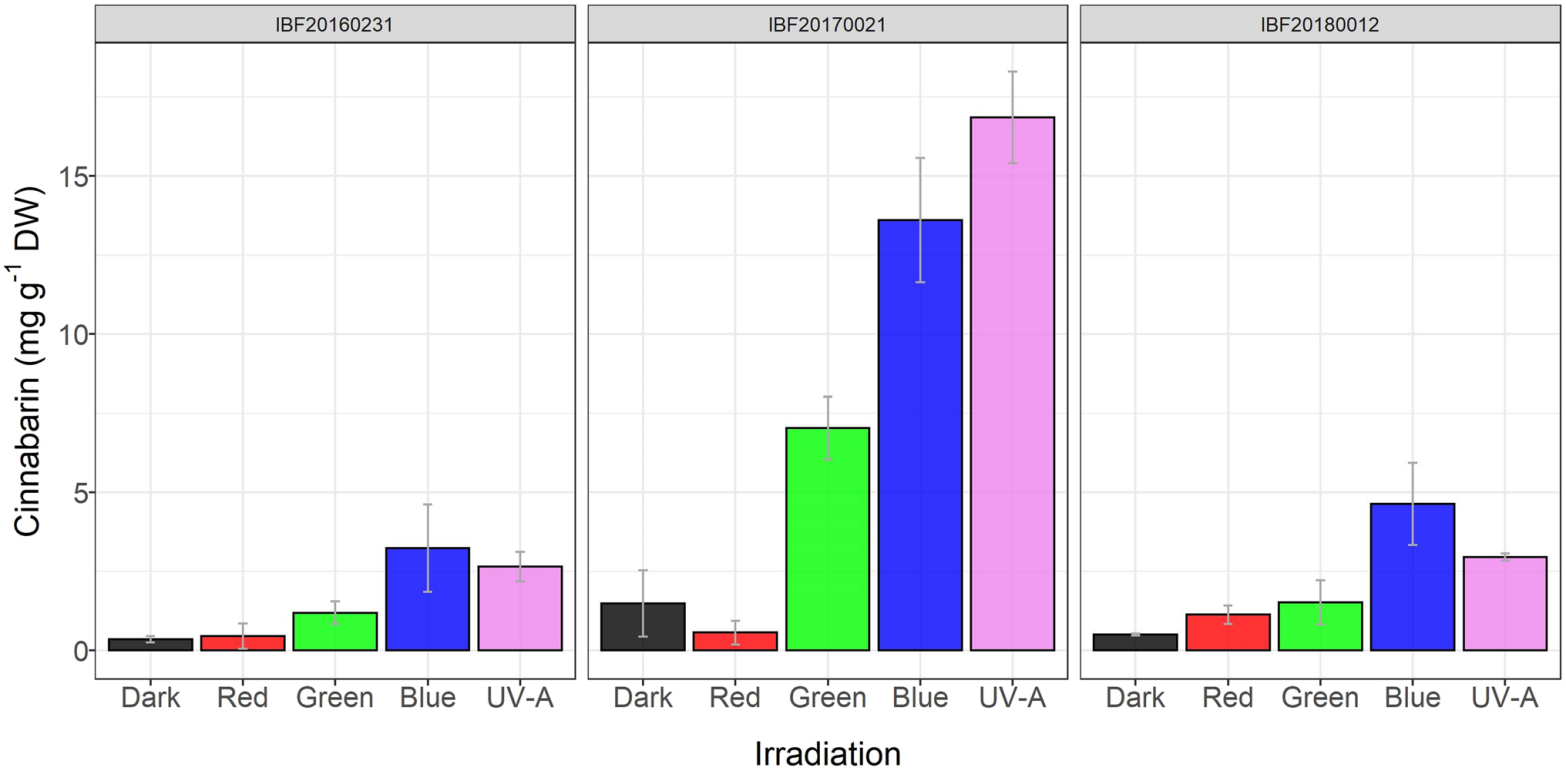
Content of cinnabarin (mg g^−1^ DW, mean sd) in cultures of three *P. cinnabarinus* strains (IBF20160231, IBF20170021, and IBF20180012) grown on MEA (7 days at 25 °C) in dependence of the following continuous irradiation conditions. Wavelength of peak emission, *λ*_peak_, and FWHM are indicated in brackets: red (635 ± 18 nm), green (519 ± 38 nm), blue (452 ± 19 nm), and UV-A (369 ± 13 nm). Average irradiance for light spectra in the center of the Petri dishes was *E*_e_ = 1.5 ± 0.18 W m^−2^

The highest cinnabarin content relative to biomass was found for strain IBF20170021, which at the same time showed the lowest growth compared to the other two strains (Figs. 2, 3). The opposite was the case for strain IBF20180012 under UV-A irradiation, which showed the highest formation of biomass and one of the lowest cinnabarin content relative to biomass.

Overall, the cinnabarin content correlated clearly with the irradiation wavelength in a species-specific pattern, i.e., blue light and UV light increased the cinnabarin content in all three strains compared to cultures grown in the dark. However, the resulting cinnabarin quantity was highly strain specific (Fig. 3).

Cinnabarin was constitutively present in all strains and its levels were generally low in cultures grown in the dark or under red light. As indicated before, the content depended on the irradiation condition and differed between the strains. Increased cinnabarin contents were detected above a certain, strain-specific photon energy. Below this energy, the content was statistically indistinguishable from the one under dark conditions. In two strains (IBF20160231 and IBF20180012), the content was slightly increased under green irradiation with respect to dark or red conditions. However, the difference was insignificant. In contrast, in strain IBF20170021, green irradiation increased the detectable cinnabarin content by a factor of 4 to 5 compared to dark or red conditions. Irradiation with high energetic blue or UV-A light increased the cinnabarin content in all strains even more.

**Fig. 4:**
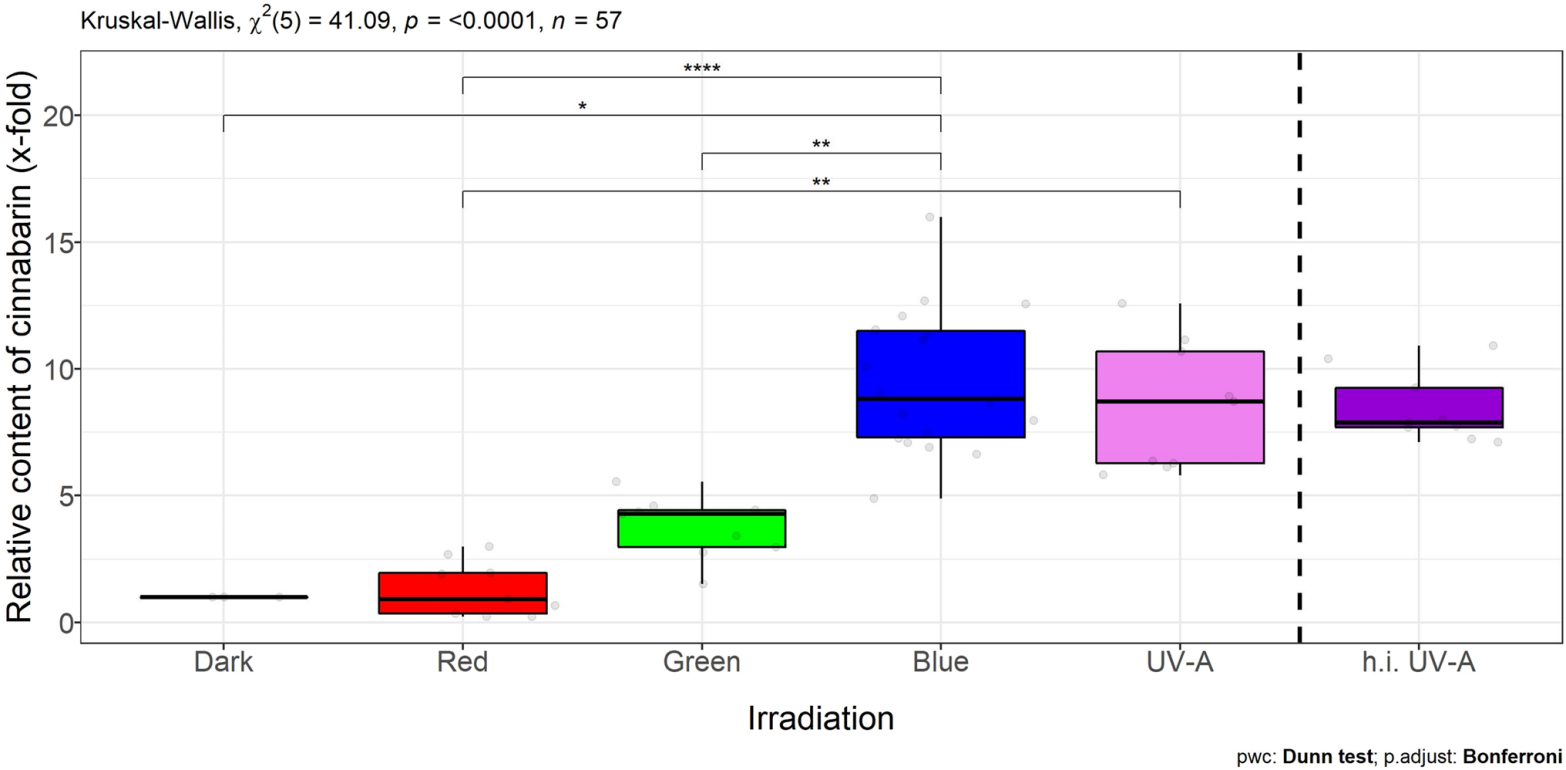
Relative content of cinnabarin (*x*-fold) with respect to content under dark or red conditions, respectively (= onefold) of three *P. cinnabarinus* strains (IBF20160231, IBF20170021, and IBF20180012) grown on MEA (7 days at 25 °C) in dependence of the following continuous irradiation conditions. Wavelength of peak emission, *λ*_peak_, and FWHM are indicated in brackets: red (635 ± 18 nm), green (519 ± 38.1 nm), blue (452 ± 18.7 nm), and UV-A (369 ± 13 nm). Irradiance in the center of the Petri dishes was *E*_e_ = 1.5 ± 0.18 W m^−2^ for all irradiations, except for higher intensity (h.i.) UV-A (369 ± 13 nm) irradiance: *E*_e_ = 5.6 ± 1.8 W m^−2^. Dark conditions served as control for light treatment or base line for the constitutive content of cinnabarin, respectively. Median and standard deviation are depicted, the light gray circles represent individual measurements of cultures

To answer the question, if the boost of the cinnabarin content under blue or UV-A irradiation was in the same order of magnitude for all strains, the contents were expressed relatively to the cinnabarin content of the respective strain under dark conditions (Fig. 4). This normalization indicated that blue or UV-A irradiation enhanced the content of cinnabarin in all strains, i.e., species specific, by a factor of 9–10 on average. Interestingly, however, the intensity did not have any further effect: irradiation with a four times more intense UV-A light did not change the content of cinnabarin (Fig. 4, h.i. UV-A).

### Culture characteristics under irradiation

*Pycnoporus cinnabarinus* exhibited quite characteristic features in pure culture, and several of the micro-morphological characteristics were photo-induced. As these cultures were obtained from fruiting body tissues, the initial mycelial growth was represented by dikaryotic, hyaline generative hyphae with clamp connections at the septa. The diameter of generative hyphae ranged from 2.5 to 4.0 µm.

Generative hyphae differentiated either into unbranched, thick-walled skeletal hyphae (diameter 2.5–4.0 µm), or into arthroconidia. Skeletal hyphae are usually unbranched and thick-walled hyphae without clamp connections. They are formed by hyaline generative hyphae gradually transforming into pigmented, thick-walled hyphae. When exposed to natural light conditions, hyphae of all transitional morphological stages produce conspicuous, extracellular, incrusted pigments forming irregular bands and crusts on the hyphal surface. These epihyphal pigments could be dissolved in 3% KOH and were highly concentrated in older cultures, where they were also present as very dark reddish brown, amorphous matter situated between the hyphae.

**Fig. 5:**
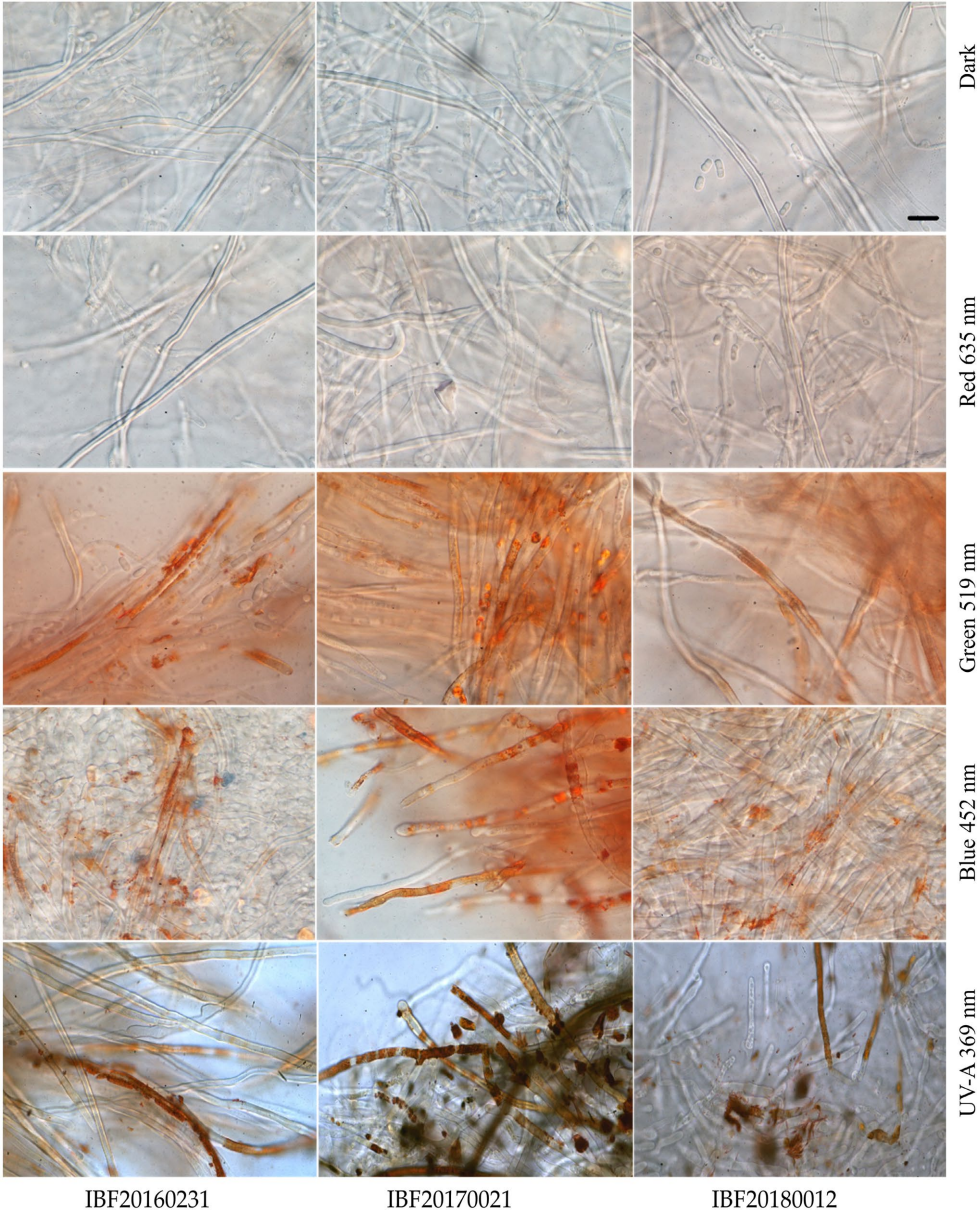
Extracellular aggregation of pigments, band-like increased at the cell wall, in dependence of different irradiation conditions (bright field microscopy, 1000-fold magnification). The scale bar equals 10 µm. Irradiance in the center of the Petri dishes was *E*_e_ = 1.5 ± 0.18 W m^−2^ for all irradiations, except for UV-A (369 ± 13 nm): *E*_e_ = 5.6 ± 1.8 W m^−2^

The observed intensity of hyphal pigment production (Fig. 5) and coloration of pure cultures was wavelength dependent, and consistent with our results from the light-dependent cinnabarin quantification. Under blue and ultraviolet irradiation, the pigmentation of the cultures was elevated (Figure S15). In contrast, it was very weak or absent under red light or in the dark.

**Fig. 6:**
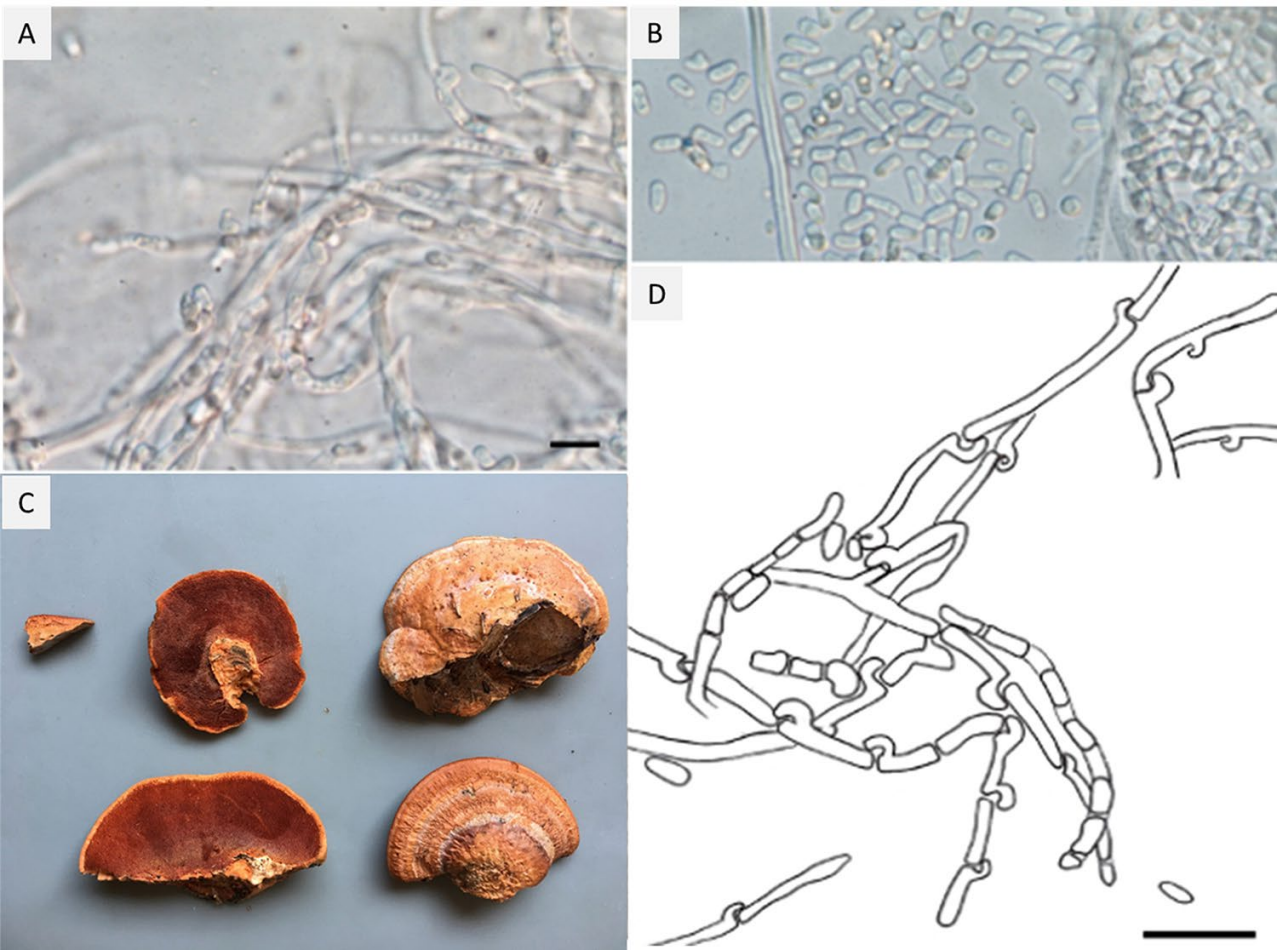
*Pycnoporus cinnabarinus.* **A** Formation of unpigmented arthroconidia in pure cultures of strain IBF20180012 and **B** strain IBF20160231. **C** Fruiting body of *Pycnoporus cinnabarinus* IBF20160231. **D** Drawing of arthroconidia formation. **A**, **B**, **D**: scale bar represents 10 µm

Arthroconidia are vegetative fungal propagules typically produced by segmentation of a pre-existing fungal hypha. Arthroconidia produced in *P. cinnabarinus* pure cultures (Fig. 6) were cylindrical or slightly irregular in shape, with varying length (4–10 µm), and a diameter of 2.5–3.5 µm resembling the diameter of the original hypha. Formation of arthroconidia was most conspicuous in unpigmented parts of the mycelium, arthroconidia are hyaline, and not a single pigmented arthroconidium was observed.

## Discussion

### Temperature range of growth

At a first glance, the observed high temperature range and growth up to 42 °C were surprising for a polypore. This observation is, however, in line with earlier reports [34]. Furthermore, the related species *P. sanguineus* has already been reported even as a rare initially causative agent of allergic bronchopulmonary mycosis [35]. The unusually high temperature optimum for growth and the thermotolerance up to 42 °C could be an adaptation to the typical habitat of these fungi: the fruiting body formation of *P.* *cinnabarinus* often occurs in particularly sunny places on warm and light-exposed deadwood. *P. cinnabarinus* is widespread on hardwood in Central and Southern Europe, but comparatively rare in Northern Europe [15].

### Estimation of fungal biomass

Some cultures exhibited an inhomogeneous distribution of hyphae in pure cultures. For example, a non-circular shape or a denser mycelial growth in random areas was observed (supplementary information, Figure S12–15). This was also true for more or less circular shaped colonies, which sometimes formed a considerable amount of aerial hyphae in random areas. Thus, the culture diameter did not provide the required accuracy as an estimate for fungal growth or biomass.

We, therefore, used dry weight as estimation for the fungal biomass, allowing us to normalize the cinnabarin content more adequately. Different methods for estimating fungal biomasses have been mentioned in the literature, such as (i) melting or cooking of agar plates [36], (ii) the use of alternative and physiologically inert gelling agents [37], (iii) analysis of metabolic biomarkers (e.g., ergosterol [38]) or (iv) mycelial growth on a cellulose hydrate membrane. The last method, using a cellophane membrane for the separation of biomass and medium, has been used for normalization [39, 40] and worked well for biomass estimation in this study. First, by simply shearing the fresh biomass from the cellophane membrane, the mycelium could be harvested quickly and completely. Second, reliable dry weight data, and thus the basis for pigment content determination, could be obtained by subsequent lyophilization of the biomass. Nevertheless, a careful preparation prior to inoculation is necessary. In detail, preparation worked best when cellophane discs were (i) individually separated in a stack by discs of white printer paper (80 g m^−2^), and (ii) embedded in a Petri dish without deionized water for autoclaving. This procedure resulted in only slightly wrinkled cellophane discs, which could be applied flat on the surface of the solidified medium without trapping air bubbles. This is important, as air bubbles can cause inhomogeneous nutrient availability, resulting in reduced fungal growth.

### Standardized irradiation conditions

Light is a well-known factor in triggering fungal growth and secondary metabolite formation [6]. It is, therefore, paramount to study these effects under clearly defined and reproducible irradiation conditions during cultivation. These requirements were met with our recently developed and custom-built irradiation system, the LIGHT BOX [41]. This device includes a light-tight ventilation system for accurate temperature and humidity homoeostasis within a climate chamber. The aim of this study was to investigate the relation between pigment content and wavelength. To ensure maximum comparability, the same irradiance (*E*_e_ = 1.5 ± 0.18 W m^−2^) was chosen for all wavelengths. This specific value was selected based on the maximal irradiance of the LEDs with the lowest power, i.e., the green one. In addition to this low irradiance, a 3.7-fold higher irradiance (i.e., *E*_e_ = 5.6 ± 1.8 W m^−2^) was selected for the UV-A irradiation to better simulate the conditions at higher altitudes. The LIGHT BOX was developed specifically for a highly standardized photo-treatment of solid-state cultivations. As fungal growth on and in solid substrates is common in natural habitats—and therefore generally physiologically advantageous for the formation of secondary metabolites [42]—, solid culture techniques are considered the technique of choice for obtaining secondary metabolites [43].

### Micro-morphological adaptations—irradiation effects on fungal growth and cinnabarin content

To distinguish possibly different effects of distinct irradiations on growth and content of cinnabarin, different spectra (*λ*_peak_ (FWHM) = 635 (18) nm, 519 (38) nm, 452 (19) nm, and 369 (13) nm, i.e., red, green, blue, and UV-A) were applied. For comparability of the impact, the irradiance was standardized to *E*_e_ = 1.5 ± 0.18 W m^−2^. Surplus, near-visible UV-A with an elevated irradiance of *E*_e_ = 5.6 ± 1.8 W m^−2^ was tested to elucidate a possible dosage dependency of UV-A irradiation and content of pigments.

Formation of biomass depended on the applied wavelengths; strain-specific positive or negative effects were observed (Fig. 2). The growth-promoting and growth-inhibiting effects of the respective wavelengths were different for each strain and did not follow a common pattern, except for UV-A, which strongly increased biomass formation in all strains, with a most remarkable increase in strain IBF20170021.

Beside an irradiation effect on the cinnabarin content which was present in all tested strains (Fig. 3), there was a distinct difference between the single strains: interestingly, in strain IBF20170021, the irradiation with green light led to fourfold–fivefold increase of the cinnabarin content compared to red light or darkness. Furthermore, while the strain with the lowest growth (IBF20170021) showed the highest relative cinnabarin content, this indirect proportion was reversed for strain IBF20180012 under UV-A irradiation (Figs. 2, 3). For fungi, such strain-specific responses are widely found in literature, [44–46]. In addition, also the correlation between growth limitation, i.e., lower biomass formation, accompanied with increased levels of primary or secondary metabolite formation are frequently documented [47–49].

Content of cinnabarin correlated, in contrast to the formation of biomass, with wavelength or irradiation energy in all three strains in a similar pattern. This response pattern was characterized by a strain-specific threshold value at different wavelengths. This threshold is a pivotal point for the effect of light on the organism. It triggered metabolic reactions at certain irradiation wavelengths. In the study, the short wavelengths (i.e., blue light or UV-A irradiation) resulted in an increased content of pigments. This indicates a possible protective function of cinnabarin against damage caused by high-energy light, or against changes in the habitat occurring synchronously along with these light dynamics.

With UV-A and blue light, both, the formation of biomass and the content of cinnabarin, were enhanced in all three strains in the same order of magnitude, with respect to dark conditions (Fig. 4). One explanation for this enhanced reaction for both irradiation conditions might be that UV irradiation and near-UV irradiation activate blue light photoreceptors such as cryptochromes or the white-collar complex [7]. Of further interest in this context is that previous reports suggest that cinnabarin and related compounds result from the radical scavenging reaction of 3-hydroxy anthranilic acid [50].

In addition, the aggregation of pigments and the exact location in relation to the hyphae (Fig. 5) give a further indication of the possible function or even the mode of action: a pigment which is extracellularly accumulated on the hyphal wall could serve as a light absorbing sunscreen, i.e., being light protective. In the lichen *Xanthoria parietina* [51], UV protection by absorption has been suggested for the extracellularly crystallized mycobiontic pigment parietin.

During vegetative growth within the woody substrate, hyphae are usually not exposed to light. However, irradiation becomes a prominent environmental factor as soon as hyphae reach areas close to the substrate surface. Precisely under these conditions, the cinnabarin content increases. Thus, the possible functions might be as diverse as the changed conditions: for example, an elevated fitness (i) in competitive interactions with other microorganisms (antifungal or antibiotic chemical defense) or macro-organisms (such as fungivore repellents in fungus–fungivore interactions), (ii) in reproductive mechanisms (e.g., transition from vegetative to sexual reproduction), and (iii) for hyphal growth.

Compared to the formation of extrolites (e.g., exoenzymes such as cellobiose dehydrogenase, laccase, or protease) in the related species *P. sanguineus*, the cinnabarin content was increased by opposite irradiation conditions. In detail, the production of extrolites was highest in the absence of light and only partially inhibited by red light, whereas it was reduced by > 85% under green and blue light irradiation [52]. On the other hand, the cinnabarin content increased due to blue light and U-A irradiation. These opposite reactions to light make sense, as substrate degrading enzymes are only needed as long as the mycelium is growing in the respective substrate, thus in the dark.

### Irradiation effects on micromorphology

The transition from vegetative growth to a reproductive survival strategy is usually induced by changes in the availability—or the properties—of the substrate and the surrounding habitat. For example, vegetative growth and highly efficient vegetative reproduction might be preferred within the nourishing substrate, i.e., in the dark [50]. In contrast, at the surface of a substrate, conditions including nutrient availability, temperature, water activity, and irradiation may change dramatically, forcing physiological adaptations for dispersal and survival.

The formation of arthroconidia represents a widely neglected anamorphic stage, or form of asexual reproduction of this polypore species [53]. For a white-rot fungus like *P.* *cinnabarinus,* the formation of such dikaryotic vegetative spores (see Fig. 6) could be considered as an additional, very efficient dispersal strategy: arthroconidia represent a fungal dispersal strategy based on wind, wood-inhabiting insects, or animal vectors, e.g., woodpeckers [54].

## Conclusion

In recent decades, it has become increasingly clear that light is an important factor influencing fungal physiology. The spectral composition and intensity of sunlight constantly change within the diurnal cycle of the sun and, together with the rhythmic alternation of day and night, leads to pronounced light dynamics. These constantly changing light conditions cause important metabolic responses, e.g., by imposing a circadian rhythm, or by triggering striking physiological changes necessary for a successful transition from a life phase occurring in the dark to a life phase occurring at light exposure. In the polypore *P. cinnabarinus*, such transitions are marked by changes in the growth characteristics, in reproductive structures, and in the production of chromophoric extrolites such as cinnabarin.

We showed in this study, that the content of cinnabarin correlates with the wavelength of irradiation: red light or the absence of light failed to increase the content of cinnabarin. Irradiation with blue or UV-A light increased cinnabarin content in all strains by a factor of 9 to 10, whereas green light did so only in one strain. This indicates that the threshold of the triggering wavelength is strain specific. Light is an important factor affecting fungal productivity and metabolite yield. For efficient biotechnological applications, light should be critically evaluated in advance at the level of wavelength.

### Supplementary Information

Below is the link to the electronic supplementary material.

Supplementary file1 (DOCX 7052 KB)

## Data Availability

Data available on request.
