# Supplementary material for: Growth, morphology, and formation of cinnabarin in Pycnoporus cinnabarinus in relation to different irradiation spectra
Source: Photochem Photobiol Sci. 2023 Oct 28;22(12):2861–75. doi: 10.1007/s43630-023-00493-3 (PMC10709268; doi:10.1007/s43630-023-00493-3)
Supplement: Supplementary file 1 — Supplementary file1 (DOCX 7052 KB) [file 43630_2023_493_MOESM1_ESM.docx]

Schinagl *et al.* 2023 Growth, morphology and formation of cinnabarin in *Pycnoporus cinnabarinus* in relation to different irradiation spectra

**Supplementary material section**

**Supplementary material 1** – Chemical characterisation based on original recorded spectra.

5.2121

Figure S1. HPLC-DAD chromatogram of the purified compound at a detection wavelength of λ = 254, 366, and 450 nm. The applied gradient is depicted as blue line. The detailed method is given in the experimental section.

 Figure S2 Calibration curve of cinnabarin based on the dilution of two stock solutions.


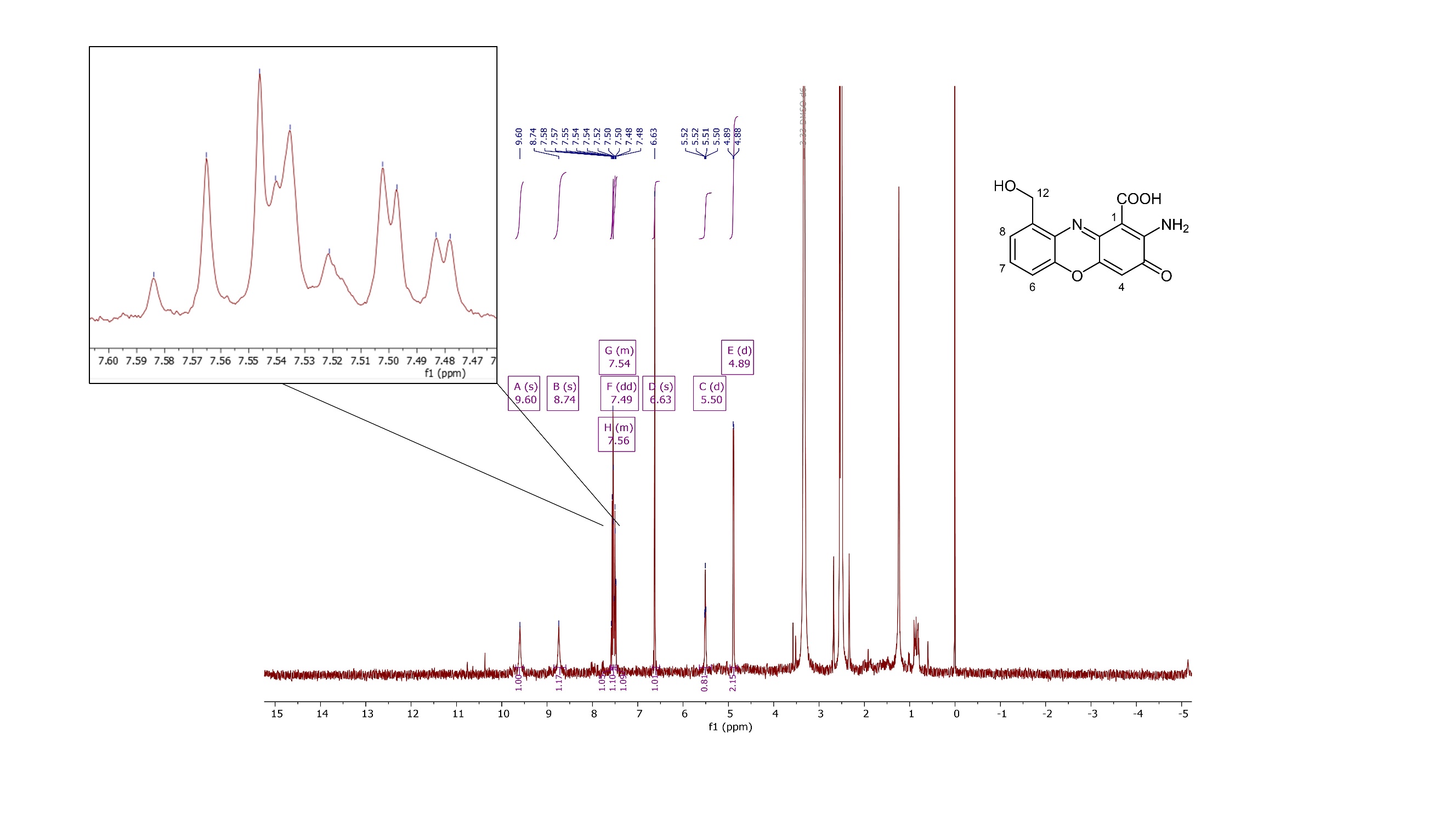


Figure S3. 1H-NMR (400 MHz, 25 °C, DMSO-d6) spectrum of cinnabarin.


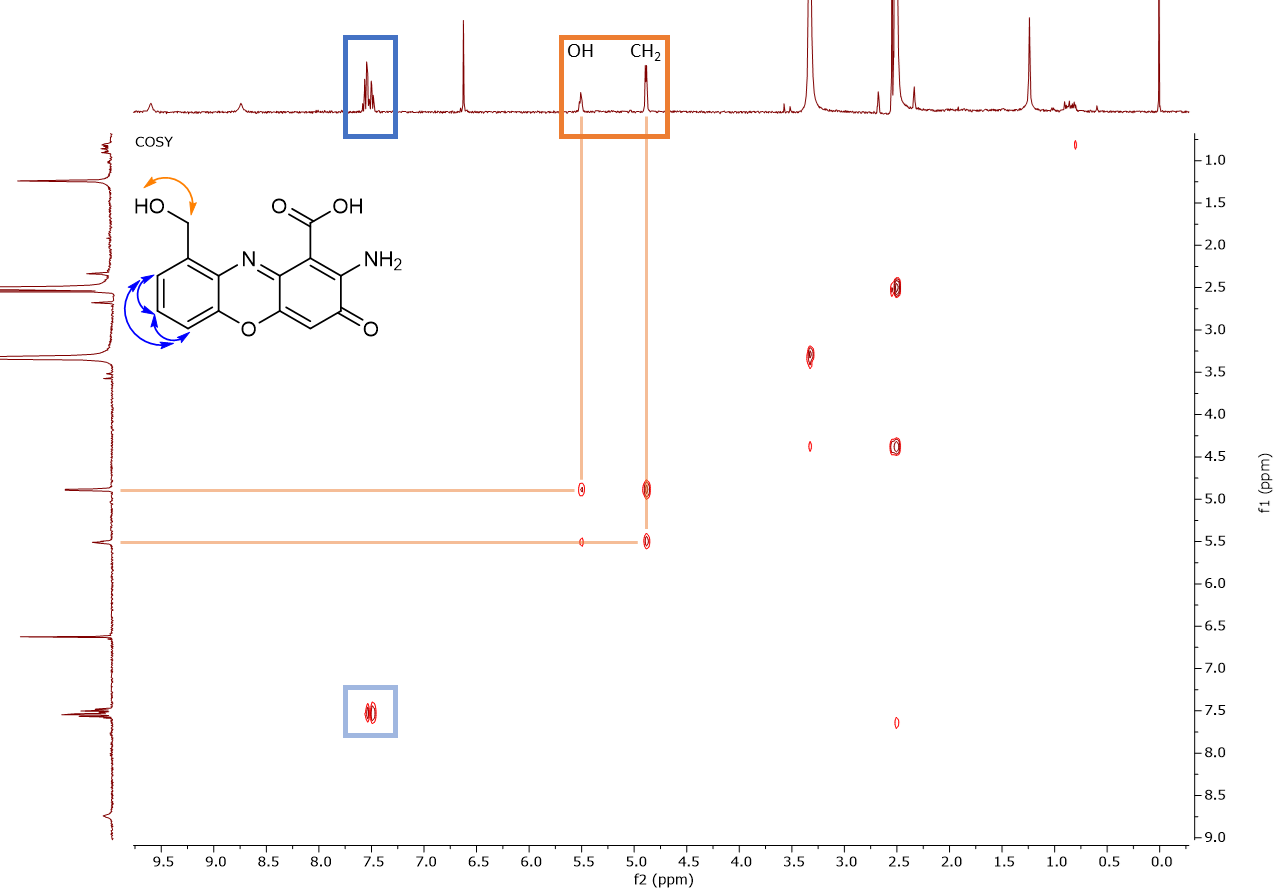


Figure S4. COSY spectrum of cinnabarin (400 MHz, 25 °C, DMSO-d6) including key correlations.

Figure S5. FT-IR-spectrum of cinnabarin.

Results Annotation Process

Figure S6. UHPLC chromatogram of the Pycnoporus cinnabarinus acetone extract, and two examplary samples #19, and sample #29 at a detection wavelength of λ = 468 nm. Cinnabarin corresponds to the peak with a retention time of 3.26 min.

Figure S7. UHPLC chromatogram of the Pycnoporus cinnabarinus acetone extract, sample #19, and sample #29 at a detection wavelength of λ = 519 nm. Cinnabarin corresponds to the peak with a retention time of 3.26 min.

Figure S8. Total ion chromatogram (TIC) of the Pycnoporus cinnabarinus acetone extract, sample #19, and sample #29 recorded in positive ionization mode. Cinnabarin corresponds to the peak with a retention time of 3.26 min.

SIRIUS results:


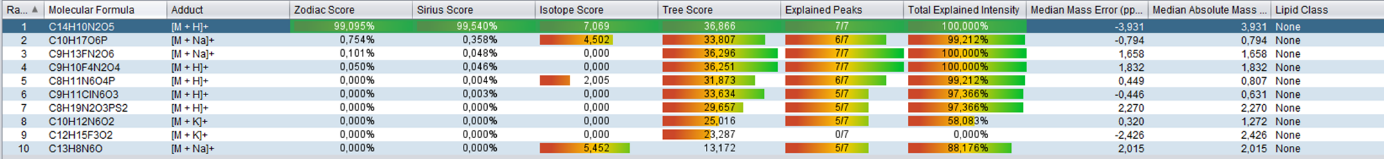


Figure S9. Result of the molecular formula prediction for the feature representing cinnabarin. The formula was predicted as C14H10N2O5.


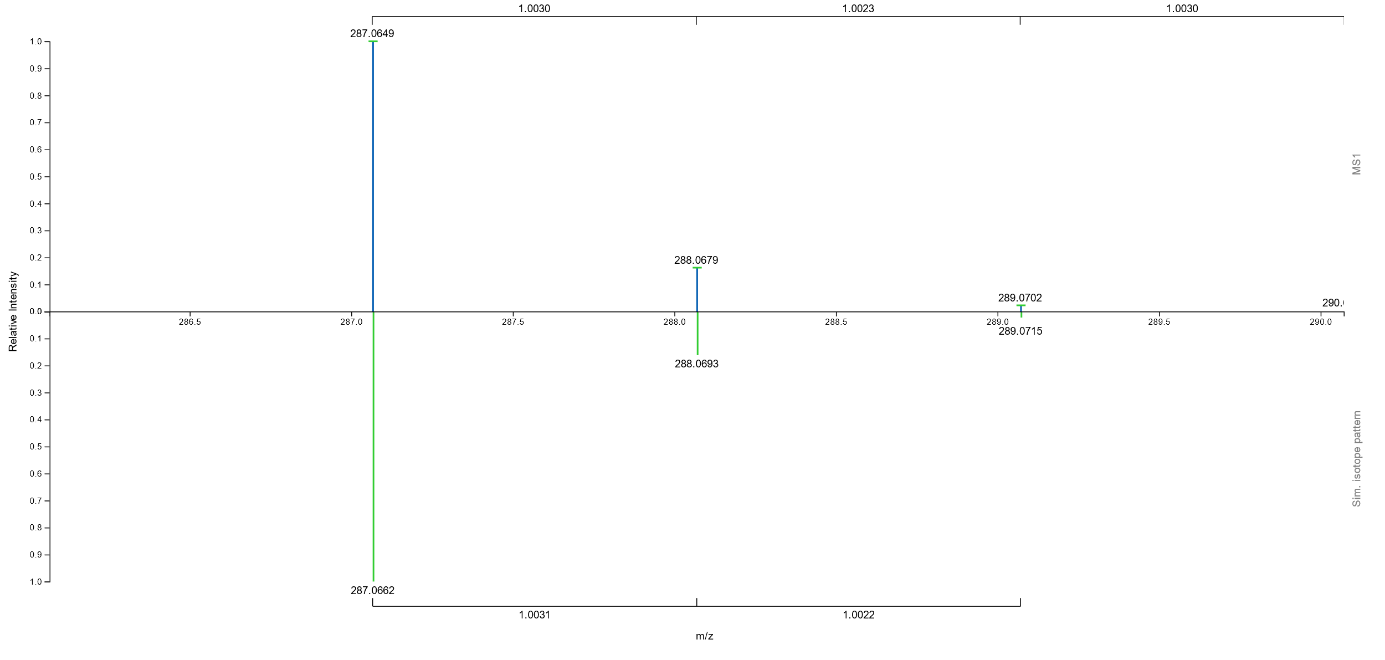


Figure S10. MS1 mirror-plot of cinnabarin (direct export from SIRIUS). The [M+H]^+^ ion is found at m/z = 287.0649.


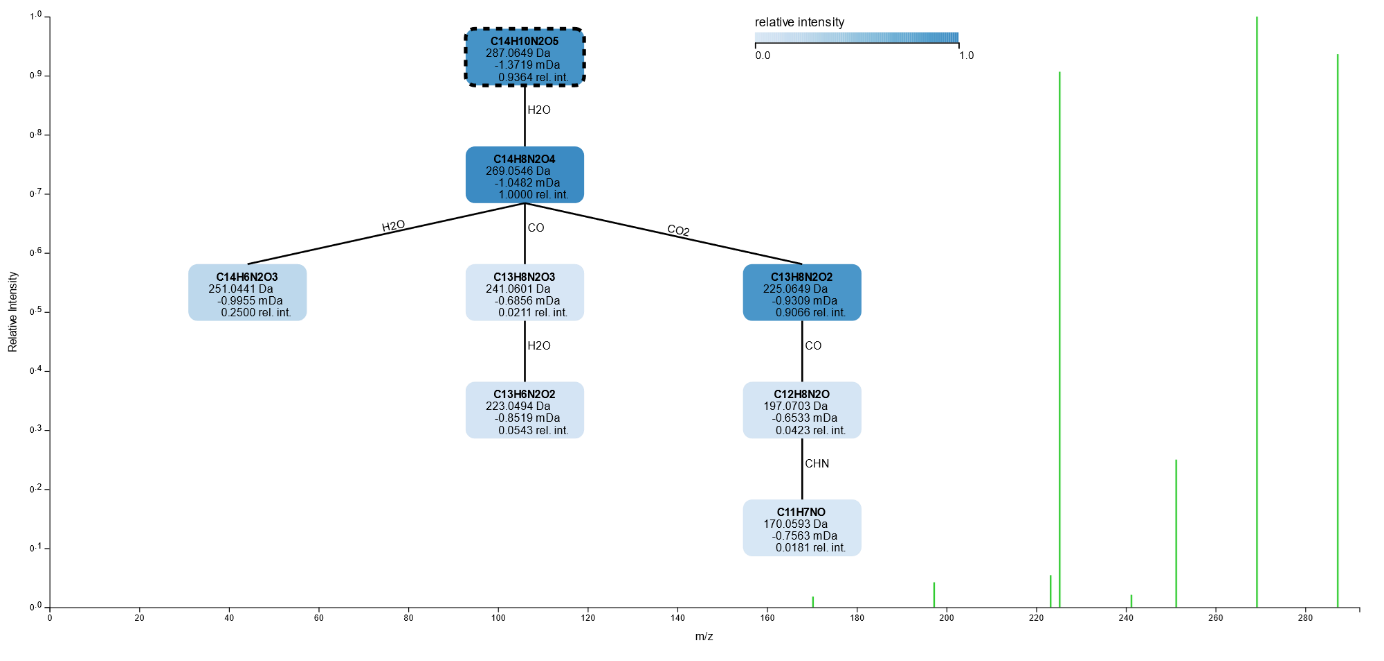


Figure S11. MS2 spectrum and the corresponding fragmentation tree of cinnabarin (direct export from SIRIUS).

Canopus results:

Table S1. Summary of Canopus results for cinnabarin given as class and probability.

| **Main Classes [Probabilities]** | | **Natural Product Classes [Probabilities]** | |
| --- | --- | --- | --- |
| Level 6 | Amino acids (97 %) | Class | Phenoxazine alkaloids (50 %) |
| Level 5 | Amino acids and derivatives (96 %) | Superclass | Anthranilic acid alkaloids (89 %) |
| Subclass | Amino acids, peptides, and analogues (96 %) | Pathway | Alkaloids (93 %) |
| Class | Carboxylic acids and derivatives (99 %) |  |  |
| Superclass | Organic acids and derivatives (100 %) |  |  |
| Kingdom | Organic compounds (100 %) |  |  |

**Supplementary material 2** - Depiction of cultures for estimation of the optimum temperature for growth and pigmentation in this study.


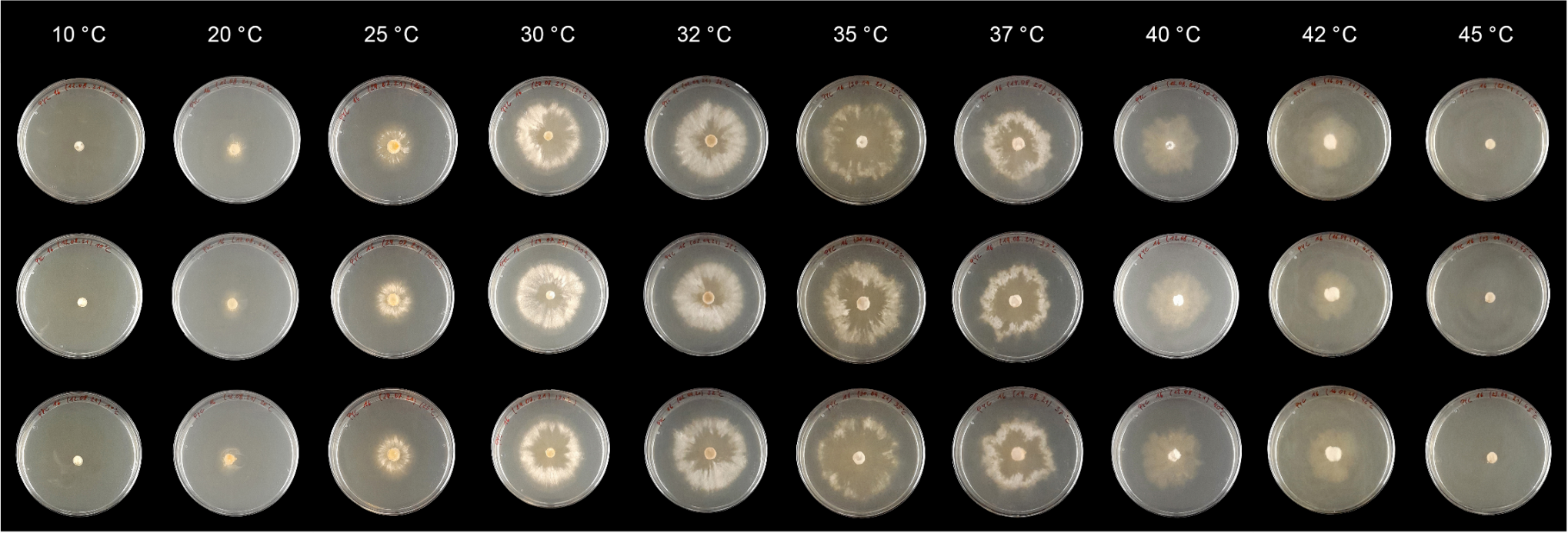


Figure S12. *P. cinnabarinus* strain IBF20160231: triplicates cultivated on MEA for seven days at different temperatures in the dark.


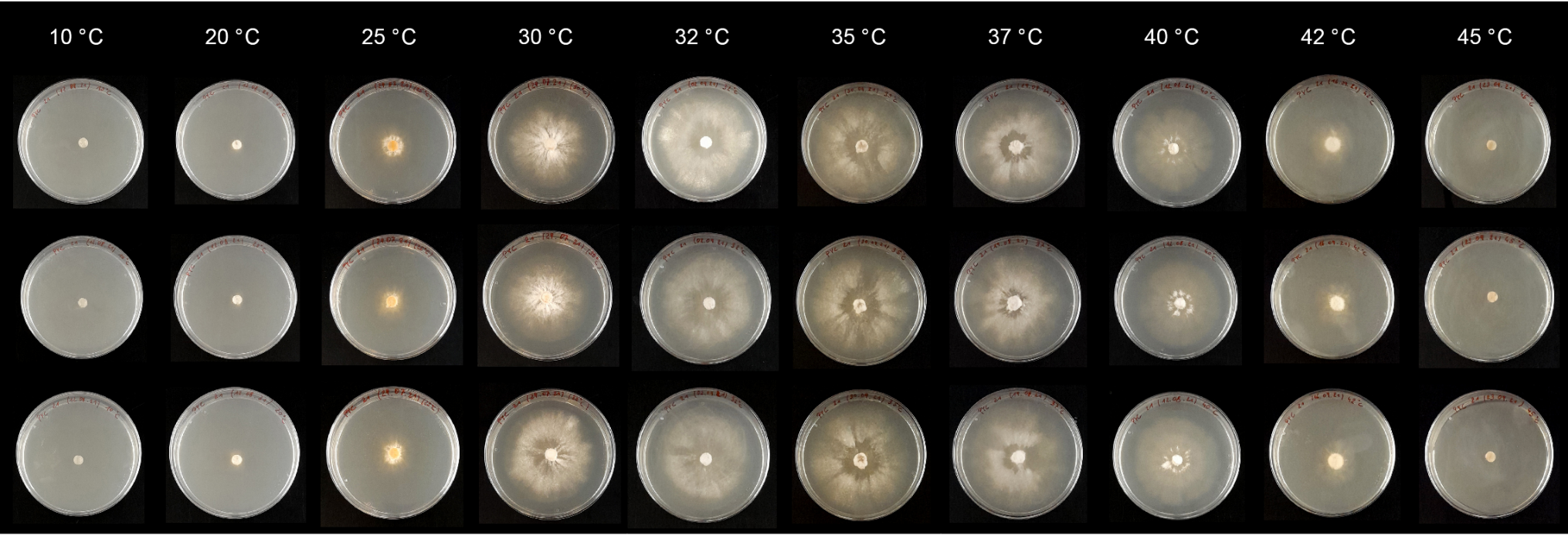


Figure S13. *P. cinnabarinus* strain IBF20170021: triplicates cultivated on MEA for seven days at different temperatures in the dark.


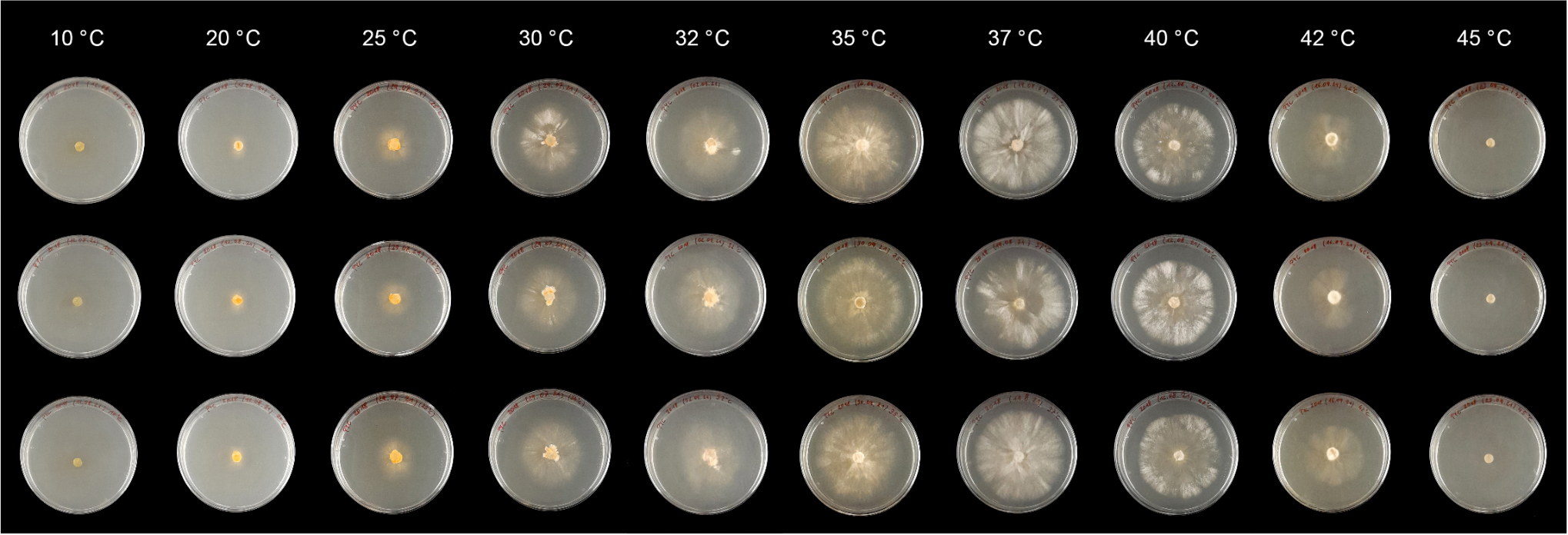


Figure S14. P. cinnabarinus strain IBF20180012: triplicates cultivated on MEA for seven days at different temperatures in the dark.

**Supplementary material 3** - Depiction of representative cultures irradiated with different spectra.


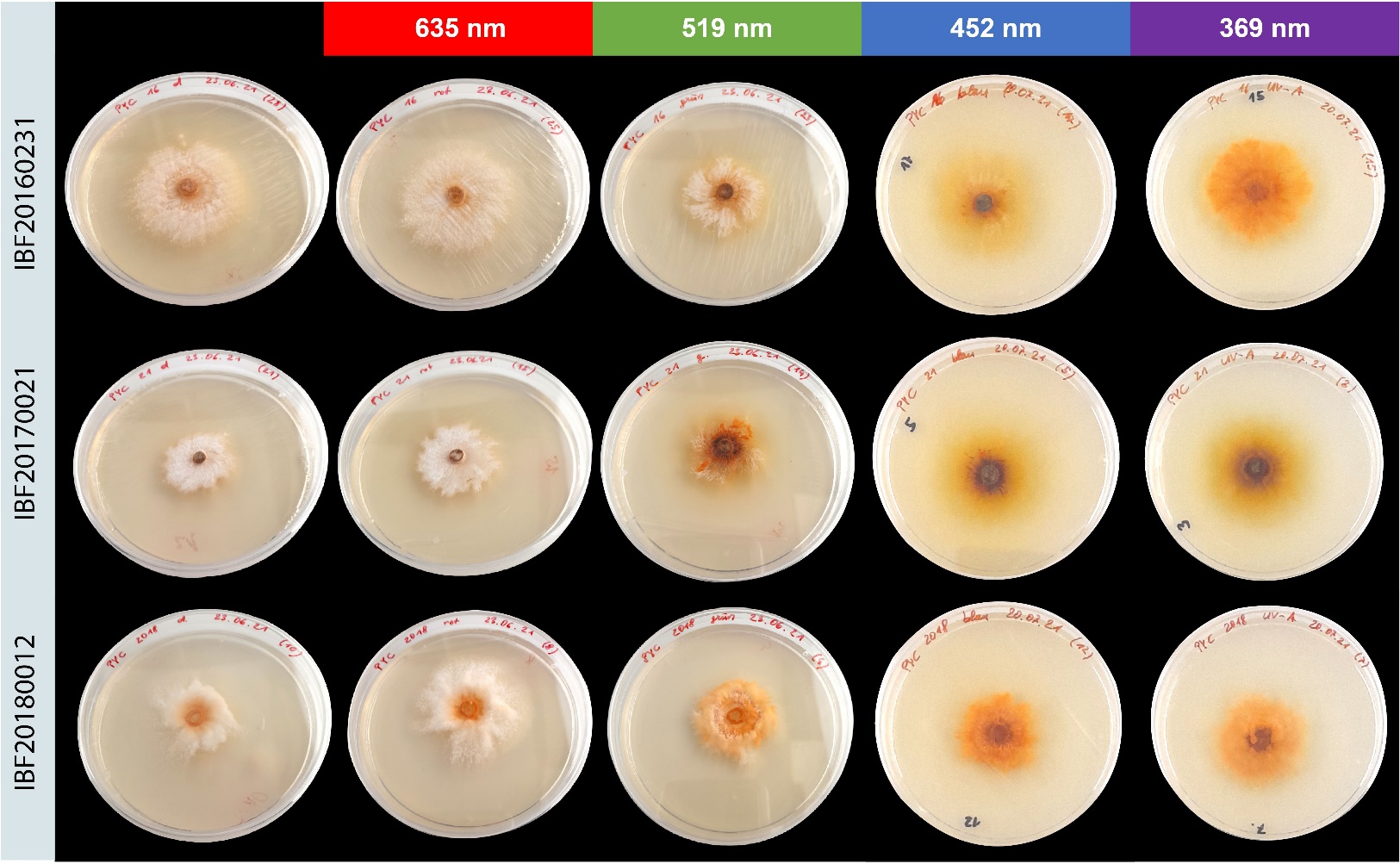


Figure S15. Representative Petri dish cultures of P. cinnabarinus (strain IBF20160231, IBF20170021, and IBF20180012) cultivated for seven days on MEA at 25 °C under different continuous irradiation conditions. Wavelength of peak emission, λ_peak_, and FWHM are indicated in brackets: red (635 ± 18 nm), green (519 ± 38.1 nm), blue (452 ± 18.7 nm), and UV-A (369 ± 13 nm). Irradiance for visible light in the center of the Petri dishes was Ee = 1.5 ± 0.18 W∙m^-2^, except for UV-A irradiance was Ee = 5.6 ± 1.8 W∙m^-2^. Dark conditions (left column) served as control.
